# Impact of Low Lithium Concentrations on the Fatty Acids and Elemental Composition of *Salvinia natans*

**DOI:** 10.3390/molecules28145347

**Published:** 2023-07-11

**Authors:** Anamaria Iulia Török, Ana Moldovan, Lacrimioara Senila, Eniko Kovacs, Maria-Alexandra Resz, Marin Senila, Oana Cadar, Claudiu Tanaselia, Erika Andrea Levei

**Affiliations:** 1 Research Institute for Analytical Instrumentation Subsidiary, National Institute of Research and Development for Optoelectronics INOE 2000, 67 Donath Street, 400293 Cluj-Napoca, Romania; iulia.torok@icia.ro (A.I.T.); eniko.kovacs@icia.ro (E.K.); alexandra.resz@icia.ro (M.-A.R.); marin.senila@icia.ro (M.S.); oana.cadar@icia.ro (O.C.); claudiu.tanaselia@icia.ro (C.T.); erika.levei@icia.ro (E.A.L.); 2 Faculty of Horticulture, University of Agricultural Sciences and Veterinary Medicine, 3-5 Manastur Street, 400372 Cluj-Napoca, Romania

**Keywords:** *Salvinia natans*, elemental composition, lithium exposure, photosynthetic pigments, fatty acids

## Abstract

The photosynthetic pigments, protein, macro and microelements concentrations, and fatty acids composition of *Salvinia natans*, a free-floating aquatic plant, were analyzed after exposure to Hoagland nutrient solution containing 1, 3, and 5 mg/L Li. The Li content of *Salvinia natans* grew exponentially with the Li concentration in the Hoagland nutrient solution. The exposure to Li did not induce significant changes in Na, Mg, K, Cu, and Zn content but enhanced the Ba, Cr, Mn, Ni and Mo absorption in *Salvinia natans*. The most abundant fatty acids determined in oils extracted from *Salvinia natans* were C16:0, C18:3(*n*6), C18:2(*n*6), and C18:3(*n*3). The photosynthetic pigments did not change significantly after exposure to Li. In contrast, chlorophyll and protein content decreased, whilst monounsaturated and polyunsaturated fatty acids content increased after the exposure to 1 mg/L Li. The results indicated that *Salvinia natans* exposed to low Li concentrations may be a good source of minerals, omega 6 and omega 3.

## 1. Introduction

Aquatic plants have a fast growth rate, high photosynthetic efficiency, good biomass productivity and high content of protein, starch, sugar, and fat [1]. Based on their characteristics, aquatic plants are a promising feedstock source, suitable for producing bioresources for protein, fish and other animal feed or supplements, soil compost, biofertilizers, biofuels and other value-added products [1].

*Salvinia natans* (L.) All. (*S. natans*) is a native species in central and southeastern Europe. It has a high growth rate and reproduction capacity under various environmental stresses. Moreover, it is easy to spread and harvest and can remove organic and inorganic contaminants from aqueous environments [2]. These properties make *S. natans* ideal as test plants for phytoremediation and biofortification [2]. Moreover, in some countries, *S. natans* is used as food due to its high nutritional value [3]. In literature, there are several studies on the heavy metals (Cu, Cd and Zn) and Cr(VI) removal from aqueous medium by *S. natans* [4]. *S. natans* is also used for cooking oil removal from aqueous solutions [3]. Casas and Matamoros (2022) showed an increase of long-chain compounds in *S. natans* structure under exposure to different concentrations of micropollutants (naproxen, diclofenac, carbamazepine, and benzotriazole) [5].

Lithium (Li) has different functions in the growth and development of plants. It may have a beneficial biofortification role in plant metabolism. However, it is highly dependent on the plant species. An addition of 5 mg/L of Li was found to increase biomass productivity by fixing the plant’s organic matter [6]. Generally, low concentrations of Li (≤12 mg/L) increase productivity, sugar, ascorbic acid, citric and malic acid level in fruits [7]. Moreover, it enhances CO_2_ fixation and positively affects the carbohydrate and nitrogen metabolism, respiratory activity, chlorophyll, nucleic and organic acid content of plants [6].

Butterhead lettuce (*Lactuca sativa*) increased the fresh roots’ weight following exposure to low (2.5 mg/kg) Li concentrations in soils. Likewise, a stimulatory effect on the root systems elongation was observed [8,9]. Exposure to 20 mg/L Li decreased by 20% the lettuce biomass 50 mg/L Li led to a severe decrease in biomass (≈70%), while 100 mg/L Li completely blocked the plant growth [8]. Maize also showed significant growth after being exposed to 5 mg/L Li [10]. Tobacco (*Nicotiana tabacum*) plants treated with 30–50 mM Li induced the formation of necrotic spots and leaf curling, as well as incompatible pathogen interactions [11]. A 30 m M Li exposure had no negative effect on cabbage (*Brassica carrinata*) seedling germinations [12] but affected its chlorophyll, lipid and phenolic composition [9,12]. Li react with unsaturated fatty acids and destroy the lipid bilayer [13]. Halophilic plants, such as *Carduus arvense* and *Holoschoenus vulgaris*, have a high tolerance and accumulation capacity (100–230 µg/g) [8,14]. These results suggest that Li concentrations above 20 mg/L have a negative effect on plants’ physiological and biochemical responses. However, this threshold is species-dependent, and the effect of Li on the plant’s chemical composition is not fully understood. In the present study, a domain of 1 to 5 mg/L Li concentrations was considered to investigate the stimulating effect on *S. natans,* as no previous studies regarding the Li stimulating effect on aquatic plants were found. The plants’ vital signs were monitored according to the photosynthetic pigment’s variation after Li exposure.

In recent years, fatty acids (FAs) have been used as bioindicators for evaluating aquatic plants’ exposure to pollutants [15]. FAs are essential cell membrane molecules and can participate in numerous processes, such as cellular transport, permeability, and metabolic partway.

*S. natans* is a source of monounsaturated fatty acids (MUFA) and polyunsaturated fatty acids (PUFA). The change in fatty acids and elemental compositions of *S. natans* in response to different concentrations of Li has not been reported in the literature. However, some studies indicate the effect of Li on lipid oxidation [16]. The exposure of *Lemna* and *Salvinia* to pharmaceuticals was found to influence the concentration of fatty acid components. A decrease in the concentration of myristic (C14:0), stearic (C18:0), and palmitic acid (C16:0) was reported, whereas the concentration of long-chain fatty alcohol and very long-chain fatty acids increased [5]. Bejaoui et al. (2019) used fatty acids to indicate lead chloride toxicity in *Venus verrucosa* gills. They reported a decrease in omega-3 concentrations (eicosapentaenoic acid (C20:5(*n*3)) and docosahexaenoic acid (C22:6(*n*3)) and an increase in the omega-6 fatty acids content [17]. These were caused by the oxidative deterioration of polyunsaturated lipids and by the biosynthesis process in the presence of metals. The oxidation mechanism is produced by oxygen or metals [17,18].

The evaluation of FAs composition is essential in the selection of plant species as food ingredients. Lipids are classified based on the presence of double bonds in saturated fatty acids (SFAs), monounsaturated fatty acids (MUFAs), and polyunsaturated fatty acids (PUFAs). Depending on the number of carbon atoms, fatty acids can contain between 14 and 20 carbon atoms (SFAs) or more than 20 carbon atoms (PUFAs). Thus, SFAs do not have double bonds, MUFAs contain one double bond, and PUFAs contain two or more double bonds. PUFAs can be arranged in the cis or trans isomeric form [19]. A high PUFA content is essential for food nutrition. The most important PUFAs are α-linolenic acid (C18:3(*n*3)) and linoleic acid (C18:2(*n*6)). Although *S. natans* properties and composition were widely investigated, there are few studies on their fatty acid composition. Rozentsvet et al. (2005) analyzed fatty acids in *S. natans* oil using a mixture of solvents, CHCl_3_:CH_3_OH: C_6_H_6_:NH_4_OH (130:60:20:12), and reported the presence of 16:1, 18:1, 20:1 and 18:3 fatty acids [20]. Based on published data, the fatty acid content of *Salvinia* species depends on the sub-species and culture conditions. The efficiency of lipids extraction depends on the method used, solvent, and types.

Generally, oil extraction from *Salvinia* species can be carried out by conventional methods with solvents. The aqueous plants must be sufficiently dry for the cells to break and release the oil during solvent extraction. It can be carried out in static or dynamic (Soxhlet extractor) mode. In literature, the solvents used to extract plant oil were mostly hexane, petroleum ether, methanol, ethanol, and chloroform. However, new extraction alternatives use green methods, such as solvents combined with ultrasonication, supercritical fluid extraction, pressurized solvents, microwave irradiation, etc. At high temperatures, PUFAs can be degraded. Ultrasound extraction is a method that uses sound waves of a frequency higher than 18–20 kHz. The ultrasound-assisted extraction method has become an alternative to conventional methods due to its high efficiency in extracting fatty acids with low molecular weight [21,22]. 

This study aimed to evaluate the impact of culture medium amendment with low Li concentrations (1, 3 and 5 mg/L) on the content of photosynthetic pigments, protein, macro- and microelements and the possible changes in the fatty acids composition of *S. natans* to verify the hypothesis that low Li concentration can enhance plant metabolism. 

## 2. Result and Discussion

### 2.1. Lithium Impact on the Plant’s Photosynthetic Pigments and Total Protein Content

**Figure 1:**
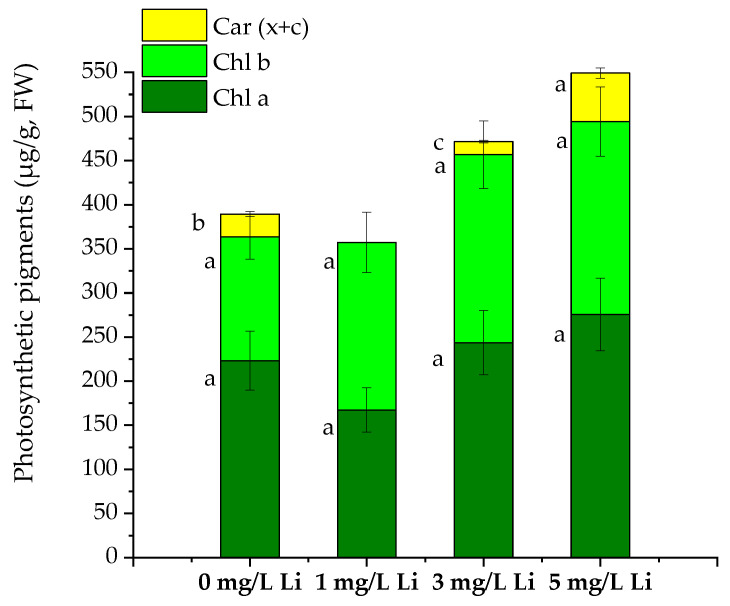
The photosynthetic pigments content (chlorophyll a—Chl a chlorophyll b—Chl b, and total carotenoids—Car (x + c)) of *S. natans* grown for seven days in control (0 mg/L Li) and in 1, 3 and 5 mg/L Li solutions. Different letters indicate different values at *p* < 0.05 significance level.

There are physical and chemical factors that influence plant growth. Thus, Li and other elements can play a significant role in enhancing or blocking the plant’s development [13]. By exposure to low concentrations of Li (1–5 mg/L), the *S. natans* aquatic plants showed no statistically significant differences in the chlorophyll contents (Chl a and Chl b) (Figure 1). The Chl a content decreased (25%) after the exposure to 1 mg/L Li and increased by 9 and 20% after the exposure to 3 and 5 mg/L Li, respectively (Figure 1).

The Chl b content increased by 30 to 50% in all plants exposed to Li (Figure 1). Compared to the control plants (0 mg/L Li), the Car (x + c) content decreased to an undetectable level in the plants exposed to 1 mg/L Li, decreased by 42% in those exposed to 3 mg/L and doubled in the case of the plants exposed to 5 mg/L Li. The correlation analysis showed a high correlation of Li concentration with Chl a (r = 0.74) and Chl b (r = 0.87) and a moderate correlation with Car (x + c) (r = 0.69) content.

Previously, it was reported that the sunflower chlorophyll a and b contents were not affected. Only the carotenoids decreased after Li supplementation (25 mg/L Li) [10]. While other species showed a reduction in the Chl a and b contents after the exposure to Li (in hydroponic conditions), there are species, such as spinach (in soil medium), that showed an increase in the Chl b content after Li exposure [23,24].

The C and H content did not differ significantly after the Li exposure. However, a very small increase of the C and H content was observed in the case of *S. natans* exposed to 1 mg/L Li, compared to the control plants (Table 1). The S content was below 0.01% in all studied samples. Previous studies revealed that *S. natans* has a high growth rate and a high biomass production and, therefore, a high nutrient uptake rate in nutrient-rich water [25]. Thus, in the present study, the small variation of the elemental composition of *S. natans* after the Li treatment showed that a small concentration of Li does not interfere with the *S. natans* growth and development.

*S. natans* showed no significant changes in their protein content (%, DW) after the exposure to Li, except for the exposure to 1 mg/L Li, where a decrease of 30% was observed. Therefore, it can be concluded that a low level of Li exposure does not induce additional stress in *S. natans*. Many other elements were reported to induce the protein oxidation process. Cd and Ni were found to inhibit protein synthesis due to their possible bond with the protein sulfhydryl groups (PSH) [27,28]. Moreover, *S. natans* exposed to Al presented a protein oxidation rate 2.72 times higher than in the control plants. Exposure of *S. natans* to toxic compounds resulted in the loss of proteins in functional states due to protein oxidation, decline of protein synthesis and acceleration of protein turnover [29]. Previous studies reported that glutamic acid, aspartic acid and glycine are the most abundant non-essential amino acids, whereas leucine, lysine, arginine and valine are the most abundant essential amino acids in aquatic plants [7,30]. 

High correlations were found between protein and Chl a (r = 0.88) and Car (x + c) (r = 0.88) contents, while there was no correlation between Chl b and protein contents.

### 2.2. The Li Impact on the Macro and Micro Elements Content in S. natans

**Figure 2:**
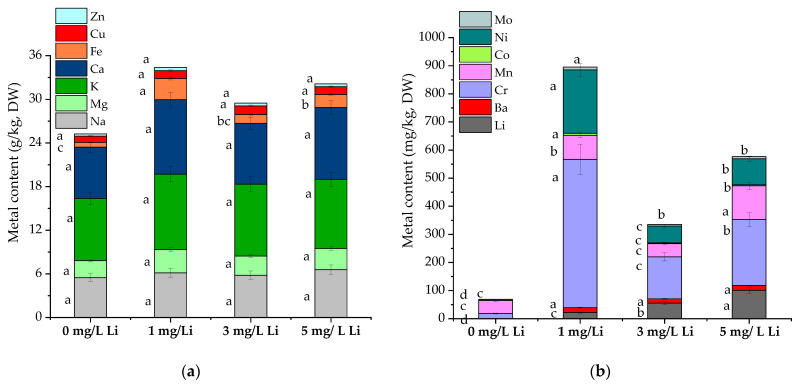
The *S. natans* macro- and microelement content after a Li supplementation with 0, 1, 3 and 5 mg/L: (**a**) Macroelement content: Na, Mg, K, Ca, Fe, Cu and Zn (g/kg DW); (**b**) Microelement content: Li, Ba, Cr, Mn, Co, Ni, Mo (mg/kg, DW). Different letters indicate different values at *p* < 0.05 significance level.

The Li content in *S. natans* was enhanced gradually by increasing the Li concentrations in the Hoagland nutrient solution; the exposed plants accumulated 21.7, 55.4, and 101 mg/kg (DW) Li, compared to the control experiments (Figure 2). 

Fe’s contents were 1.9–4.4-fold greater and significantly differed from the control content (0 mg/L Li). Na and Li contents were positively correlated (r = 0.81), indicating a strong relationship. Previous studies showed that Li has both stimulatory and depressive effects on the concentration and uptake of other elements. Naeem et al. (2021) reported a positive correlation between Li and Na in aquatic environments [6]. Moreover, Li was reported as a substitute for other important monovalent cations (like Na and K) in plant cells and as a competitor with the alkali metals in the plant’s root uptake [31]. Ryzymski et al. (2017) found that, in the case of medicinal mushrooms, *Agrocybe cylindracea* and *Hericium erinaceus*, Li had no significant effect on the uptake of Ca, K, Mg or Na macroelements, after biofortification experiments with Li [32]. Previously, it was reported that Li had a different impact on several plant nutrients, such as Ca and K. However, in the case of lettuce and watercress, the 2 mM Li exposure showed no significant changes in the plants’ K concentrations [6]. Moreover, spinach grown in Li-enriched soil medium showed no changes in the Ca, Mg, Na, Mn, Fe Zn and Cu contents, while the K content decreased significantly [6].

In the present study, it was observed that the treatment with Li induced the uptake of essential (Ba, Mn, Mo) and nonessential (Cr, Ni) elements from the Hoagland nutrient solutions (Figure 2b), compared to the control plants (exposed to 0 mg/L Li). Interestingly, *S. natans* exposed to 1 mg/L Li showed 2–3.5-fold higher Cr content compared to the plants exposed to 3 or 5 mg/L Li. The same pattern was also noticed in the case of Ni, where *S. natans* exposed to 1 mg/L Li resulted in 2.5–3.8-fold higher Ni contents than the plants exposed to 3 or 5 mg/L Li. This data suggests that low Li contents may stimulate the absorption of microelements in *S. natans*. Exposure of *S. natans* to 5 mg/L Li showed the highest Mn content. No correlation between the Li and Cr, Ni and Co contents was found, however, moderate to high correlations were found between Li and Ba (r = 0.65) and Mn (r = 0.70).

Watanabe et al. (2016) stated that certain vegetable crop species accumulate more nonessential elements than essential macro elements. For example, Chinese chive leaves, contained high concentrations of V, Cr, Ba, Fe and Ni [33]. 

The plant species generally contain Ni in a very low concentration (0.05–10 mg/kg DW) [34]. Nickel has a key role in enzyme activities, cellular redox state, biochemical and physiological growth responses, and activation of ureas in higher plants [34,35]. Thus, a Ni deficiency reduces the activity of the ureas, resulting in necrosis and chlorosis on leaf tips and older leaves [34]. Ni is required in small quantities in some leguminous crops for nodule growth and activation of hydrogenase enzyme [34]. On the other hand, excess Ni decreases the uptake and transport of elements such as Fe, Cu, Zn, and Mn in many plant species [34].

In the present study, it was observed that *S. natans* normal micro- and nonessential elements accumulation can be disturbed by Li, and compared to the control plants, the exposure to 1 to 5 mg/L Li resulted in alternation of the Ni and Cr accumulation.

### 2.3. Lithium Impact on S. natans Fatty Acids

The composition of fatty acids of *S. natans* oil after exposure to different concentrations of Li is presented in Table 2. According to Mubarak et al. (2016), among the pre-treatments used in lipids extraction from *S. molesta* oil, ultrasonication gives the highest oil yield compared to microwaving, glass grinding, sand grinding, and autoclaving [36]. In the present study, the obtained oils yield was 5 ± 0.4%. The SFAs identified in *S. natans* oil are: myristic acid (C14:0), palmitic acid (C16:0), heptadecanoic acid (C17:0), stearic acid (C18:0), arachidic acid (C20:0), and lignoceric acid (C24:0). C16:0 is the predominant fatty acid found in all oil samples (approx. 17.3%), followed by arachidic acid (C20:0) (11.9%).

The MUFAs identified in *S. natans* are: palmitoleic acid (C16:1(*n*9)), cis-10-heptadecenoic acid (C17:1) and oleic acids (C18:1(c + t)(*n*9)). Oleic acid (C18:1(c + t)(*n*9)) is the most abundant MUFA component. A small trace of C17:1 was found in the control samples. *S. natans* had considerably greater omega-6 and omega-3 PUFA concentrations, with a high content of C18:3(n-6) (12.81%), C18:2(cis + trans)(n-6) (7.99%), and C18:3(n-3) (6.15%). Among omega-6, linoleic acid ((C18:2(c + t)(*n*6), PUFA) was the most abundant, followed by arachidonic acid (C20:4(*n*6)(PUFA). C18:3(*n*3) is the only fatty acid present in omega-3 PUFA. PUFAs benefit health by protecting the brain and preventing or mitigating cardiovascular disease due to their anti-oxidant, anti-inflammatory, anti-tumoral and anti-coagulant properties. The human body and the fish cannot synthesize two fatty acids, namely C18:2 and C18:3(*n*3), so they must be supplemented through food [37]. The PUFAs content revealed that *S. natans* is a good source of omega-6 and omega-3. According to Prado et al., the presence of some metals during the growth of *S. natans* can cause a change in photosynthesis and respiration, leading to structural alteration and possible PUFA modification [38]. The content of SFAs, MUFAs and PUFAs found in *S. natans* after exposure to different concentrations of Li is presented in Table 2. The saturated fatty acid content slightly decreased after treatments with different concentrations of Li. It was found that the MUFAs level increased in the treatment with 1 mg/L Li. The PUFAs concentration also increased after the treatments with Li. The content of C18:3(*n*6) increased after the treatment with 1 mg/L Li and decreased after the treatment with 3 and 5 mg/L Li. The content of C17:0 and C17:1 was found only in control *S. natans*, while after the treatment with Li, these two compounds were not identified.

Although some plant species can tolerate increased Li concentrations, in general, high concentrations of Li in the substrate used to grow plants were reported to have a negative impact on the roots and leaves [39,40].

The synthesis of PUFAs mainly consisted of the fatty acid synthesis pathway (FAS) and polyketosynthase (PKS) pathway. Desaturase and elongate enzymes play an important role in synthesising monounsaturated fatty acids in plants [41].

Increased Li content can inhibit enzyme activities, reducing plant growth [42]. On the other hand, Li at low concentrations can stimulate plant growth. In this work, low Li amounts were added to improve plant growth. As shown in Table 2, the omega-6 content increased when 1 mg/L Li was added to the Hoagland nutrient solution. Li in plants interacts with enzymes requiring Ca and Mg [10]. Li is a cofactor for the functioning of some enzymes in plants. It may appear as a replacement factor to improve metabolism due to its affinity to enzymes activated by Ca or Mg [13]. However, the beneficial effects of Li on the *S. natans* fatty acids contents still require further investigation to expand the knowledge related to the dose-dependent response. 

### 2.4. Cluster Analysis

**Figure 3:**
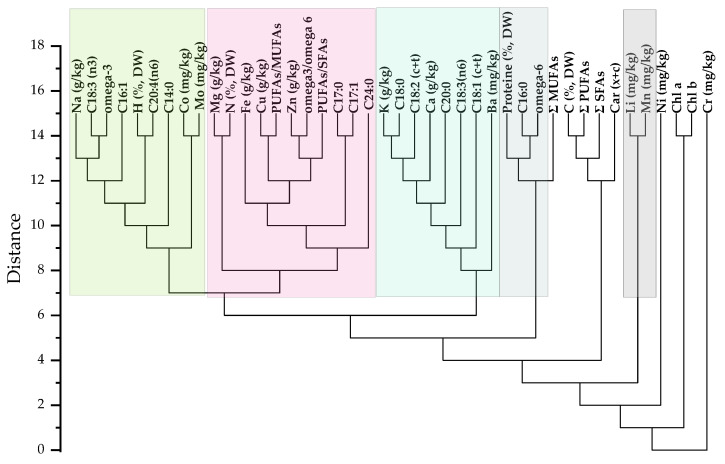
Dendrogram on the data variables of *S. natans* after the exposure to 0, 1, 3 and 5 mg/L Li concentrations in Hoagland nutrient solutions.

A grouping of Na with C18:3(*n*3) and omega 3 was observed (Figure 3). Alpha-linolenic acid (C18:3(*n*3)) is a short-chain polyunsaturated fatty acid from the omega-3 family, which proves the strong correlation. The Pearson correlation between C18:3(*n*3) and C16:0(1.0) was very high, as well as with C18:0 (0.97) (Table S2). A high correlation was obtained between C18:2(c + t) and Cu (r = 0.92) and chl b (0.99) and C18:1(c + t) (r = 0.90). This suggests that Cu can enhance the activity of plants to accumulate more Li. A significant correlation between fatty acids C18:1 and C18:2 (r = 0.90) was shown. A very high correlation was obtained between MUFA and Mg (r = 1.0), Ca (r = 0.97), Fe (r = 0.99), Zn (r = 0.93), Cr (r = 0.98), Co (r = 0.99), Ni (r = 0.97), Mo (r = 0.96) and moderate negative correlation with saturated fatty acid. 

The omega 3/omega 6 ratio showed a moderate correlation only with C16:0, whereas PUFA/MUSA showed a positive correlation with C16:0 and C18:3(*n* − 3). The same result was reported by Carvalho et al. for *Artemisia* spp. plant [43]. The presence of essential minerals helps plants grow and maintain their proper physiological properties.

Low Li concentrations (1, 3 and 5 mg/L Li) did not significantly affect the *S. natans* major element content, such as Na, Mg, K, Ca, Cu and Zn but enhanced the Fe uptake. Exposure to Li had a significant synergetic effect on the uptake and accumulation of minor essential elements, such as Ba, Cr, Mn, Ni and Mo, compared to the control plants. Mo has a major role in Fe metabolism, while Mo and Fe play important roles in symbiotic nitrogen fixation during plant growth and in different life stages to full maturity [44]. According to Wysokinski et al. (2022) [44], the bioaccumulation factors of Mo and Fe in peas (*Pisum sativum* L.) are the highest in the initial growth stages. These results are consistent with the current study, indicating that Li can enhance Fe and Mo accumulation in *S. natans* (in the growing period of 30–40 days) and may help the plants’ metabolism in symbiotic nitrogen fixation. 

### 2.5. FT-IR Analysis

**Figure 4:**
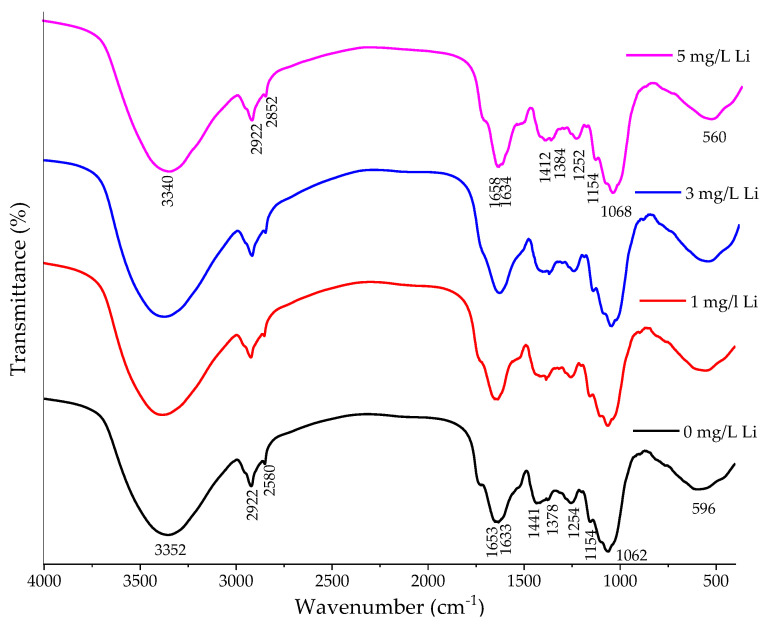
FT-IR spectra of *S. natans* exposed to 0, 1, 3 and 5 mg/L Li concentrations.

The FT-IR spectra (Figure 4) of *S. natans*, after exposure to 0, 1, 3 and 5 mg/L Li, were similar and indicated that the addition of Li had no negative effects on the structure and composition of *S. natans*. 

The strong band at 3340–3380 cm^−1^ was attributed to the O–H stretching in phenols, alcohols, or alkaloids. The bands around 2920 and 2850 cm^−1^ are specific to aliphatic C–H stretching vibrations [45]. The large band observed at 1632–1658 cm^−1^ is specific to the aromatic C=C vibrations [46]. The small peak in 1733–1734 cm^−1^ could be attributed to the C=O stretching vibration of lignin, whereas the peak at 1519–1523 cm^−1^ was due to the aromatic skeletal vibrations of the lignocellulose contents. The peaks at 1412–1441 cm^−1^ and 1378–1384 cm^−1^ were attributed to stretching vibrations of guaiacyl rings and C–O in lignin [46]. The large peak at 1062–1068 cm^−1^ was attributed to the C–O stretch in polysaccharides, cellulose, and hemi-cellulose, while a large, broad band at 556–569 cm^−1^ to C=O bend vibrations [47].

## 3. Materials and Methods

### 3.1. Experimental Design

*S. natans* aquatic plants were obtained from a local aquarium store in Cluj-Napoca, Romania and were grown and multiplied for 30–40 days at a temperature of 19–24 °C, under natural light conditions, in a Hoagland nutrient solution containing: 1.25 mM KNO_3_, 1.25 mM Ca(NO_3_)_2_, 0.5 mM MgSO_4_, 0.25 mM KH_2_PO_4_, 10 μM FeEDTA, 11.6 μM H_3_BO_3_, 4.5 μM MnCl_2_ 4H_2_O, 0.19 μM ZnSO_4_ 7H_2_O, 0.12 μM Na_2_MoO_4_·2H_2_O, 0.08 μM CuSO_4_·5H_2_O [45], supplemented with 0.10 μM BaCl_2_, 0.08 μM NiSO_4_∙6H_2_O, 0.08 μM Cr_2_(SO_4_)_3_, and 0.04 μM CoCl_2_∙6H_2_O. The nutrient solutions were supplemented with low concentrations of non-essential elements (Ba, Ni, Co, and Cr), which water could contain in trace concentrations due to natural sources. For the experiments, plants with a growing age of 30–40 days were used. *S. natans* were exposed to Hoagland solution enriched with 1, 3 and 5 mg/L Li, prepared from a 1 g/L stock solution, obtained by dissolving LiSO_4_·H_2_O in Hoagland nutrient solution. The plants were exposed to Li-enriched solutions [45] for seven days under laboratory conditions, following the method of Torok et al. (2022). For the control experiments, a Hoagland nutrient solution without Li addition was used (0 mg/L Li). 

### 3.2. Chemicals and Reagents

LiSO_4_·H_2_O, HNO_3_ 65% (*v*:*v*), H_2_O_2_ 30% (*v*:*v*), NaHSO_4_, KCl, KOH, methanol, isooctane, and chloroform of analytic reagent grade were purchased from Merck (Darmstadt, Germany). FAME standard mixture (Supelco 37 component FAME mix, CRM47885) was purchased from Sigma–Aldrich (Darmstadt, Germany). Ultrapure water (Elga Veolia, High Wycombe, UK) was used for the dilutions and the preparation of the standard solutions.

### 3.3. Elemental Composition

After seven days of exposure to different Li concentrations, the *S. natans* plants were washed, freeze-dried using a Labconco FreeZone 2.5 L system (Labconco, Kansas City, MO, USA) and ground to powder. Fifty mg sample was digested with 5 mL of 65% HNO_3_ and 2 mL of 30% H_2_O_2_ in a closed-vessel Speedwave XPERT (Berghof, Eningen, Germany) microwave digestion system. After cooling, the digested samples were diluted with ultrapure water to a final volume of 20 mL. The measured metal concentrations were expressed as mg/kg dry weight (DW). The Na, Mg, K, Ca, Fe Mn, Cu, Zn and Li concentrations were measured using an Optima 5300 DV inductively coupled plasma–atomic emission spectrometer (ICP–OES, Perkin Elmer, Waltham, MA, USA), while the Ba, Cr, Mn, Co, Ni, and Mo concentrations were determined using an ELAN DRC II inductively coupled plasma–mass spectrometer (ICP–MS, Perkin Elmer, Waltham, MA, USA). The instruments were calibrated using ICP multi-element standard solution IV, 1000 mg/L (Merck, Darmstadt, Germany), and multi-element Calibration Standard 3 (Perkin Elmer Pure Plus, Waltham, MA, USA), while the measurement accuracy was tested using the standard reference material, 1643f NIST–Trace elements in water (National Institute of Standards and Technology, Gaithersburg, MD, USA) and NIM–GWB 10019 Apple–Trace elements (Institute of Geophysical and Geochemical Exploration, Langfang City, China). The detection limit of each element measured by ICP-OES and ICP-MS are presented in Table S1. The average recoveries ranged between 93–104%. 

The N, C, H and S contents were determined by combustion at 1000 °C using a Flash 2000 CHNS/O analyzer (Thermo Fisher Scientific, Waltham, MA, USA) calibrated with standard BBOT (2,5-bis-(5-tertbutyl-benzoxazol-2-yl) thiophene) (Thermo Fisher Scientific, Waltham, MA, USA).

### 3.4. Photosynthetic Pigments Content Determination

The *S. natans* plants’ photosynthetic pigments (chlorophyll a—Chl a, chlorophyll b—Chl b, and carotenoids—Car (x + c)) were extracted using 0.5 g fresh weight (FW) plant biomass in 5 mL of methanol. The plant–methanol mixture was vortexed thoroughly for 1 min. and incubated at 70 °C for 3 min. The extracts were centrifuged, and the absorbance of the supernatant was measured at 665, 652, and 470 nm using a Nanodrop One Analyzer (Thermo Fisher Scientific, Waltham, MA, USA). The content of Chl a, Chl b, and Car (x + c) pigments was calculated according to Lichtenthaler (1987), and the results were expressed as µg/g (FW) [48].

### 3.5. Oil Extraction from S. natans

For the determination of fatty acids in *S. natans*, the oil was extracted using the green ultrasonic-assisted extraction method of Gasparini et al. (2023) [22] with slight modifications, as follows: 0.5 g of dried plant sample (DW) were extracted by sonication with 25 mL chloroform: methanol (2:1, *v*/*v*), using an ultrasonic bath (Bandelin Sonorex, Berlin, Germany) at 25 °C for 1 h. The biomass was separated by filtration, and the extract phase was treated with 10 mL 0.74% KCl solution. The organic phase was separated by filtration using Na_2_SO_4_ for water removal. The solvent was removed using a rotary evaporator Laborota 4010 (Heidolph, Schwabach, Germany). The obtained oil was weighed, and the oil yield was calculated. Then, the transmethylation method under alkali-catalysed conditions was applied to the obtained oil samples to convert the glycerides of the fatty acids into the corresponding methyl esters, according to Senila et al. (2020) [49]. The samples (0.06 g) were dissolved in isooctane, treated with 0.2 mL 2 M KOH solution in methanol, and vigorously stirred for 30 s. Finally, the mixture was treated with 1 g NaHSO_4_ to prevent the methyl esters’ saponification and to neutralize the excess alkali. Each oil sample was trimethylated and analysed in three replicates.

### 3.6. Instrumentation and Chromatographic Conditions

The FAMEs content was determined by GC-FID (Agilent Technologies, 6890N GC, Santa Clara, CA, USA) equipped with a ZB-WAX capillary column (30 m × 0.25 mm × 0.25 µm) and a flame ionization detector (FID, Agilent Technologies 7683, Santa Clara, CA, USA). The gas carrier was helium (purity ≥ 99.999%) with a constant 1 mL/min flow rate. The injection volume was 1 µL in 1:20 split mode. The GC oven temperature program consists of three stages: 60 °C for 1 min, 60 to 200 °C (rate 10 °C/min, kept for 2 min), from 200 to 220 °C (rate 5 °C/min, kept for 20 min). The temperature of the injector and detector was set to 250 °C. The data processing was performed with the Agilent OpenLab CDS Chemstation software C.01.10 running under Windows (Microsoft, Santa Clara, CA, USA). The FAs in samples were identified by comparing their retention time with that of the Supelco FAME standard mixtures standard mixture.

### 3.7. FT-IR Analysis

The Fourier transform infrared (FT-IR) spectra of powdered plant samples were recorded between 400–4000 cm^−1^ on KBr pellets containing 1% sample using a Spectrum BX II (Perkin Elmer, Waltham, MA, USA) spectrometer.

### 3.8. Statistical Analysis

The graphical processing of data and statistical analysis were performed using OriginLab (version 2020b, Northampton, MA, USA) software. All data were expressed as mean ± standard error, and the differences between the studied parameters were tested by comparing the averages of the three replicates using Tukey’s test for a significance level of *p* = 0.05 using Paired Comparison App (Two-Way ANOVA).

## 4. Conclusions

*S. natans* aquatic plants grown and multiplied under laboratory conditions were exposed to Hoagland solutions enriched with Li concentrations of 1, 3 and 5 mg/L Li to assess the plant’s metabolism. Regarding photosynthetic pigment content, only moderate differences were noted after exposure to Li. *S. natanss* total protein content revealed no significant changes, except for a 30% decrease after the exposure to 1 mg/L Li. The Li treatment induced a significant Ba, Cr, Mn, Ni and Mo absorption in plants compared to the control plants (exposed to 0 mg/L Li). In contrast, Li accumulation had no significant effect on the macroelements content (Na, Mg, K, Cu and Zn) of *S. natans*. All the samples presented saturated, monounsaturated, and polyunsaturated fatty acids. The most abundant fatty acids were C16:0, C18:3(*n*6), C18:2(*n*6), and C18:3(*n*3). FT-IR spectra did not reveal changes in the composition of *S. natans* exposed to various Li concentrations. The results indicate that *S. natans* is an effective source of minerals, omega 6 and omega 3 fatty acids. Data from the current study provide valuable information on the influence of Li on the fatty acids and elemental composition of *S. natans*. However, the analyses were limited to low Li concentrations. Thus, further experiments on the exposure to a wider spectrum of Li concentrations and other elements may be of interest, providing additional information on the potential application of *S. natans* in various supplements. Increasing the use of this abundant and valuable aquatic plant in different industries would be a promising solution for the food industry and for the remediation of environmental pollution.

## Figures and Tables

**Table 1:** The elemental composition and protein content of *S. natans* exposed to various Li concentrations (0, 1, 3 and 5 mg/L Li). Different letters indicate different values at *p* < 0.05 significance level.

Element (%, DW)	0 mg/L Li	1 mg/L Li	3 mg/L Li	5 mg/L Li
C	35.9 ± 1.79 ^a^	39.1 ± 1.96 ^a^	37.1 ± 1.85 ^a^	36.6 ± 1.83 ^a^
H	4.76 ± 0.24 ^a^	5.03 ± 0.25 ^a^	5.00 ± 0.25 ^a^	4.73 ± 0.24 ^a^
N	3.64 ± 0.18 ^ab^	2.57 ± 0.13 ^c^	3.26 ± 0.16 ^b^	3.78 ± 0.19 ^a^
Protein *	22.8 ± 1.14 ^ab^	16.1 ± 0.80 ^c^	20.4 ± 1.02 ^b^	23.6 ±1.18 ^a^

* The protein contents are calculated as “crude protein” = 6.25 × nitrogen content (*N*) [26].

**Table 2:** Fatty acids (FAs), saturated fatty acids (SFAs), monounsaturated fatty acids (MUFAs), polyunsaturated fatty acids (PUFAs) and PUFAs/SFAs ratio in the analyzed *S. natans* oil samples.

Fatty Acid	0 mg/L Li	1 mg/L Li	3 mg/L Li	5 mg/L Li
C14:0	5.59 ± 0.56 ^efg^	8.76 ± 0.88 ^cde^	5.13 ± 0.88 ^fg^	8.19 ± 0.82 ^def^
C16:0	17.26 ± 1.73 ^a^	16.84 ± 1.68 ^a^	18.90 ± 1.89 ^a^	16.27 ± 1.63 ^a^
C16:1(*n*9)	5.90 ± 0.59 ^fg^	5.96 ± 0.60 ^fg^	5.22 ± 0.52 ^fgh^	5.49 ± 0.55 ^fgh^
C17:0	2.61 ± 1.68 ^gh^	nd.	nd.	nd.
C17:1	2.81 ± 0.28 ^h^	nd.	nd.	nd.
C18:0	9.84 ± 0.98 ^bcd^	9.44 ± 0.98 ^cd^	10.10 ± 1.00 ^bcd^	9.42 ± 0.94 ^cd^
C18:1(c + t)(*n*9)	9.76 ± 0.98 ^de^	13.50 ± 1.35 ^ab^	15.03 ± 1.50 ^a^	12.99 ± 1.30 ^ab^
C18:2(c + t)(*n*6)	7.99 ± 0.80 ^ef^	9.93 ± 0.99 ^de^	11.19 ± 1.12 ^bcd^	11.08 ± 1.11 ^bcd^
C18:3(*n*6)	12.81 ± 1.28 ^abc^	15.06 ± 1.51 ^a^	9.97 ± 0.98 ^cde^	10.01 ± 1.12 ^cde^
C18:3(*n*3)	6.15 ± 0.62 ^fg^	6.06 ± 0.61 ^fg^	6.54 ± 0.65 ^fg^	5.88 ± 0.58 ^fg^
C20:0	11.87 ± 1.19 ^bc^	10.09 ± 1.01 ^bcd^	12.88 ± 1.29 ^b^	10.78 ± 1.08 ^bcd^
C20:4(*n*6)	5.05 ± 0.51 ^gh^	4.36 ± 0.44 ^gh^	5.05 ± 0.51 ^gh^	5.04 ± 0.50 ^gh^
C24:0	2.37 ± 0.24 ^gh^	nd.	nd.	3.36 ± 0.34 ^fg^
Σ SFAs	49.5	45.1	47.0	48.0
Σ MUFAs	24.1	28.2	25.4	26.7
Σ PUFAs	32.0	35.4	32.8	32.0
PUFAs/MUFAs	1.3	1.3	1.3	1.2
PUFAs/SFAs	0.65	0.78	0.70	0.67
omega-6	17.86	19.42	15.02	15.05
omega-3	6.15	6.06	6.54	5.88
omega3/omega 6	0.34	0.31	0.44	0.39

Data are expressed as a percentage of total FAMEs and represent mean ± standard deviation (n = 3); nd—the FA was not detected. Values indicated with different letters were significantly different from each other at *p*  ≤  0.05 levels, whereas the same letters showed no significant differences (*p* > 0.05).

## Data Availability

The data presented in this study is available upon request from the corresponding author.

## Supplementary Materials

The following supporting information can be downloaded at: https://www.mdpi.com/article/10.3390/molecules28145347/s1, Table S1: Limit of detection (LOD) of plant elements; Table S2: Pearson correlation coefficients of variables.
