# Supplementary material for: Impact of Low Lithium Concentrations on the Fatty Acids and Elemental Composition of Salvinia natans
Source: Molecules. 2023 Jul 11;28(14):5347. doi: 10.3390/molecules28145347 (PMC10385638; doi:10.3390/molecules28145347)
Supplement: Supplementary file 1 [file molecules-28-05347-s001.zip › molecules-2450907-supplementary.pdf]

Table S1. Limit of detection (LOD) of elements in plants.

**Element**	Na	Mg	K	Ca	Fe	Cu	Zn	Mn	Li	Ba	Cr	Mn	Co	Ni	Mo
**Unit**	mg/kg	mg/kg	mg/kg	mg/kg	mg/kg	μg/kg	μg/kg	μg/kg	μg/kg	μg/kg	μg/kg	μg/kg	μg/kg	μg/kg	μg/kg
**LOD**	0.0032	0.0036	0.0012	0.0016	0.0032	0.084	0.124	0.052	0.012	0.0001	0.0001	0.0001	0.0001	0.0001	0.0001

Table S2. Pearson correlation coefficients of variables.

Variable	X1	X2	X3	X4	X5	X6	X7	X8	X9	X10	X11	X12	X13	X14	X15	X16	X17	X18	X19	X20	X21	X22	X23	X24	X25	X26	X27	X28	X29	X30	X31	X32	X33	X34	X35	X36	X37	X38	X39	X40	X41	X42
**X1**	1.0																																									
**X2**	0.8	1.0																																								
**X3**	0.5	0.8	1.0																																							
**X4**	0.9	1.0	0.8	1.0																																						
**X5**	0.6	1.0	0.8	0.9	1.0																																					
**X6**	0.5	0.5	0.8	0.6	0.5	1.0																																				
**X7**	0.6	0.9	1.0	0.9	0.9	0.7	1.0																																			
**X8**	0.8	0.3	0.3	0.5	0.2	0.6	0.2	1.0																																		
**X9**	0.8	0.8	0.9	0.9	0.8	0.9	0.9	0.6	1.0																																	
**X10**	0.6	1.0	0.9	0.9	1.0	0.5	0.9	0.1	0.7	1.0																																
**X11**	1.0	0.7	0.3	0.8	0.6	0.2	0.4	0.7	0.6	0.5	1.0																															
**X12**	0.7	1.0	0.9	1.0	1.0	0.6	1.0	0.3	0.9	1.0	0.6	1.0																														
**X13**	0.5	0.9	0.9	0.9	1.0	0.4	0.9	0.0	0.7	1.0	0.5	1.0	1.0																													
**X14**	0.8	1.0	0.9	1.0	0.9	0.7	1.0	0.5	0.9	0.9	0.6	1.0	0.9	1.0																												
**X15**	0.0	-0.6	-0.8	-0.5	-0.7	-0.4	-0.8	0.4	-0.4	-0.8	0.1	-0.7	-0.8	-0.6	1.0																											
**X16**	0.3	0.8	0.9	0.7	0.9	0.5	0.9	-0.1	0.7	1.0	0.2	0.9	1.0	0.8	-0.9	1.0																										
**X17**	-0.1	0.4	0.8	0.3	0.5	0.7	0.7	-0.3	0.5	0.6	-0.3	0.6	0.6	0.5	-0.9	0.8	1.0																									
**X18**	0.0	-0.6	-0.8	-0.5	-0.7	-0.4	-0.8	0.4	-0.4	-0.8	0.1	-0.7	-0.8	-0.6	1.0	-0.9	-0.9	1.0																								
**X19**	0.3	-0.4	-0.4	-0.2	-0.5	0.1	-0.5	0.7	0.0	-0.6	0.2	-0.4	-0.6	-0.2	0.9	-0.7	-0.6	0.9	1.0																							
**X20**	0.8	0.6	0.7	0.7	0.4	0.9	0.6	0.9	0.9	0.4	0.5	0.6	0.4	0.7	-0.1	0.3	0.3	-0.1	0.4	1.0																						
**X21**	0.5	-0.2	-0.5	0.0	-0.3	-0.2	-0.5	0.7	-0.1	-0.4	0.5	-0.3	-0.5	-0.2	0.9	-0.7	-0.9	0.9	0.9	0.2	1.0																					
**X22**	0.8	0.9	0.5	0.9	0.9	0.1	0.7	0.3	0.6	0.8	0.9	0.8	0.8	0.8	-0.4	0.6	0.0	-0.4	-0.3	0.3	0.1	1.0																				
**X23**	-0.6	-0.4	0.1	-0.5	-0.4	0.4	-0.1	-0.2	-0.1	-0.4	-0.8	-0.3	-0.3	-0.2	-0.1	-0.1	0.5	-0.1	0.0	0.1	-0.4	-0.8	1.0																			
**X24**	-0.2	0.2	-0.2	0.0	0.3	-0.7	0.0	-0.7	-0.4	0.3	0.0	0.1	0.3	-0.1	-0.3	0.3	-0.1	-0.3	-0.7	-0.7	-0.3	0.4	-0.6	1.0																		
**X25**	-0.7	-0.8	-0.9	-0.8	-0.7	-0.9	-0.9	-0.7	-1.0	-0.7	-0.5	-0.8	-0.6	-0.9	0.4	-0.6	-0.5	0.4	-0.1	-0.9	0.1	-0.5	0.0	0.5	1.0																	
**X26**	-0.7	-0.8	-0.9	-0.8	-0.7	-0.9	-0.9	-0.7	-1.0	-0.7	-0.5	-0.8	-0.6	-0.9	0.4	-0.6	-0.5	0.4	-0.1	-0.9	0.1	-0.5	0.0	0.5	1.0	1.0																
**X27**	-0.8	-0.7	-0.3	-0.8	-0.7	0.1	-0.4	-0.3	-0.4	-0.7	-0.9	-0.6	-0.6	-0.6	0.2	-0.4	0.2	0.2	0.2	-0.2	-0.3	-1.0	0.9	-0.5	0.3	0.3	1.0															
**X28**	0.4	0.5	0.8	0.6	0.5	1.0	0.7	0.5	0.9	0.5	0.2	0.6	0.5	0.7	-0.4	0.5	0.7	-0.4	0.1	0.9	-0.2	0.1	0.4	-0.7	-0.9	-0.9	0.1	1.0														
**X29**	0.7	0.5	0.7	0.6	0.4	0.9	0.6	0.8	0.9	0.3	0.5	0.5	0.3	0.7	-0.1	0.3	0.3	-0.1	0.4	1.0	0.2	0.2	0.2	-0.8	-0.9	-0.9	-0.1	0.9	1.0													
**X30**	-0.2	0.3	0.2	0.2	0.5	-0.4	0.3	-0.7	-0.1	0.6	-0.1	0.4	0.6	0.2	-0.7	0.6	0.4	-0.7	-0.9	-0.5	-0.7	0.4	-0.3	0.9	0.2	0.2	-0.4	-0.3	-0.6	1.0												
**X31**	-0.6	-0.4	0.1	-0.5	-0.4	0.4	-0.1	-0.2	-0.1	-0.3	-0.8	-0.3	-0.3	-0.2	-0.2	0.0	0.6	-0.2	0.0	0.0	-0.5	-0.8	1.0	-0.5	0.0	0.0	0.9	0.4	0.1	-0.3	1.0											
**X32**	-0.6	-0.8	-0.4	-0.7	-0.8	0.1	-0.5	-0.1	-0.4	-0.8	-0.7	-0.7	-0.8	-0.6	0.4	-0.6	0.0	0.4	0.4	-0.1	0.0	-1.0	0.9	-0.6	0.3	0.3	1.0	0.1	0.0	-0.6	0.8	1.0										
**X33**	-0.2	-0.8	-0.7	-0.6	-0.9	-0.2	-0.8	0.3	-0.4	-0.9	-0.2	-0.8	-0.9	-0.7	0.9	-0.9	-0.6	0.9	0.9	0.0	0.7	-0.7	0.3	-0.6	0.3	0.3	0.5	-0.2	0.0	-0.8	0.2	0.7	1.0									
**X34**	0.3	-0.3	-0.7	-0.1	-0.4	-0.6	-0.6	0.4	-0.3	-0.5	0.5	-0.4	-0.5	-0.4	0.8	-0.7	-1.0	0.8	0.7	-0.1	0.9	0.1	-0.6	0.1	0.4	0.4	-0.4	-0.6	-0.2	-0.4	-0.7	-0.2	0.6	1.0								
**X35**	-0.4	-0.8	-1.0	-0.8	-0.9	-0.7	-1.0	0.0	-0.8	-0.9	-0.2	-0.9	-0.9	-0.9	0.9	-1.0	-0.8	0.9	0.6	-0.5	0.6	-0.5	-0.1	-0.1	0.8	0.8	0.3	-0.7	-0.5	-0.4	-0.1	0.4	0.8	0.7	1.0							
**X36**	0.7	1.0	0.9	1.0	1.0	0.5	0.9	0.3	0.8	1.0	0.7	1.0	1.0	1.0	-0.7	0.9	0.5	-0.7	-0.4	0.5	-0.2	0.9	-0.4	0.2	-0.8	-0.8	-0.7	0.5	0.5	0.4	-0.4	-0.8	-0.8	-0.3	-0.9	1.0						
**X37**	0.2	0.7	0.8	0.6	0.9	0.3	0.8	-0.3	0.5	0.9	0.1	0.8	0.9	0.7	-1.0	1.0	0.8	-1.0	-0.9	0.1	-0.8	0.6	-0.1	0.4	-0.4	-0.4	-0.4	0.4	0.1	0.8	0.0	-0.6	-1.0	-0.7	-0.9	0.8	1.0					
**X38**	-1.0	-0.8	-0.5	-0.9	-0.6	-0.5	-0.5	-0.8	-0.8	-0.5	-1.0	-0.7	-0.5	-0.8	0.0	-0.3	0.1	0.0	-0.3	-0.8	-0.5	-0.8	0.6	0.2	0.7	0.7	0.7	-0.4	-0.7	0.2	0.6	0.6	0.2	-0.3	0.4	-0.7	-0.1	1.0				
**X39**	0.3	0.8	0.9	0.7	0.9	0.5	0.9	-0.2	0.6	0.9	0.2	0.9	0.9	0.8	-1.0	1.0	0.8	-1.0	-0.8	0.3	-0.7	0.6	0.0	0.3	-0.6	-0.6	-0.4	0.5	0.2	0.6	0.0	-0.5	-0.9	-0.7	-1.0	0.8	1.0	-0.2	1.0			
**X40**	-0.3	0.3	0.1	0.1	0.4	-0.4	0.2	-0.8	-0.2	0.5	-0.1	0.3	0.5	0.1	-0.7	0.5	0.3	-0.7	-0.9	-0.6	-0.7	0.4	-0.3	0.9	0.3	0.3	-0.4	-0.4	-0.7	1.0	-0.3	-0.6	-0.8	-0.3	-0.3	0.3	0.7	0.3	0.6	1.0		
**X41**	-0.6	-0.4	0.1	-0.5	-0.4	0.4	-0.1	-0.2	-0.1	-0.3	-0.8	-0.3	-0.3	-0.2	-0.2	0.0	0.6	-0.2	0.0	0.0	-0.5	-0.8	1.0	-0.5	0.0	0.0	0.9	0.4	0.1	-0.3	1.0	0.8	0.2	-0.7	-0.1	-0.4	0.0	0.6	0.0	-0.3	1.0	
**X42**	0.1	-0.3	0.0	-0.2	-0.5	0.5	-0.2	0.6	0.2	-0.5	-0.1	-0.3	-0.5	-0.1	0.5	-0.4	0.0	0.5	0.8	0.6	0.4	-0.6	0.6	-1.0	-0.3	-0.3	0.6	0.5	0.6	-0.9	0.6	0.7	0.7	0.0	0.2	-0.4	-0.6	-0.1	-0.4	-0.9	0.6	1.0

0-0.4	Low correlation	0.4-0.7	Moderate correlation	0.7-0.9	High correlation	0.9-1.0	Very high correlation

	Variable	Unit		Variable	Unit		Variable	Unit		Variable	Unit		Variable	Unit		Variable	Unit
**X1**	Na	g/kg	**X8**	Li	mg/kg	**X15**	N	%, DW	**X22**	C14:0	%	**X29**	C18:2 (c+t)	%	**X36**	Σ MUFAs	%
**X2**	Mg	g/kg	**X9**	Ba	mg/kg	**X16**	C	%, DW	**X23**	C16:0	%	**X30**	C18:3(n6)	%	**X37**	Σ PUFAs	%
**X3**	K	g/kg	**X10**	Cr	mg/kg	**X17**	H	%, DW	**X24**	C16:1	%	**X31**	C18:3 (n3)	%	**X38**	PUFAs/MUFAs	-
**X4**	Ca	g/kg	**X11**	Mn	mg/kg	**X18**	Proteine	%, DW	**X25**	C17:0	%	**X32**	C20:0	%	**X39**	PUFAs/SFAs	-
**X5**	Fe	g/kg	**X12**	Co	mg/kg	**X19**	Chl a	µg/g, FW	**X26**	C17:1	%	**X33**	C20:4(n6)	%	**X40**	omega-6	%
**X6**	Cu	g/kg	**X13**	Ni	mg/kg	**X20**	Chl b	µg/g, FW	**X27**	C18:0	%	**X34**	C24:0	%	**X41**	omega-3	%
**X7**	Zn	g/kg	**X14**	Mo	mg/kg	**X21**	Car (x+c)	µg/g, FW	**X28**	C18:1 (c+t)	%	**X35**	Σ SFAs	%	**X42**	omega3/omega 6	-
